# Using ^13^N_2_ and PET to track *in vivo* nitrogen gas kinetics during normobaric conditions

**DOI:** 10.3389/fphys.2025.1556478

**Published:** 2025-04-01

**Authors:** Edward T. Ashworth, Ryotaro Ogawa, David R. Vera, Peter Lindholm

**Affiliations:** ^1^ Department of Emergency Medicine, University of California, San Diego, CA, United States; ^2^ Department of Radiology, University of California, San Diego, CA, United States

**Keywords:** hyperbaric medicine, radiolabeling, diving, decompression sickness, extravehicular activity

## Abstract

**Introduction:**

Decompression sickness (DCS) during extravehicular activity in space or after diving is caused by gaseous nitrogen. The pathophysiology is still not fully understood, as mechanisms of dissolved gas uptake and bubble development are challenging to study. We aimed to develop a new method using nitrogen-13 (^13^N_2_) gas *in vivo* using positron emission tomography (PET) under normobaric pressure.

**Methods:**

A single anesthetized and ventilated Sprague Dawley rat lay supine inside a PET scanner for 30 min. The rat breathed oxygen for the first 2 min, then ^13^N_2_ gas mixed with oxygen for 20 min, then oxygen alone for the final 8 min. Following the scan, a mixed blood sample was taken from the heart, while the brain, liver, femur and thigh muscle were removed to determine organ radioactivity using a gamma counter.

**Results:**

The signal intensity in the PET scanner increased from baseline (0.03) to 2–12 min (0.68 ± 0.31), and 12–22 min (0.88 ± 0.06), before reducing slightly from 22 to 30 min (0.61 ± 0.04). All organs had radioactivity when measured in the gamma counter. We confirmed that the gas decayed radioactivity in expectance with the half-life of ^13^N_2_ (R^2^ = 0.9324), and that the spectroscopy peaked just over 500 keV, suggesting no additional isotopes were present.

**Discussion:**

This study successfully demonstrated a quantitative method of tracking nitrogen gas through the body both *in vivo* using PET and *ex vivo* using a gamma counter.

## 1 Introduction

Decompression sickness (DCS) is caused by gaseous nitrogen. When the partial pressure of nitrogen increases, such as when SCUBA diving to depth, tissues become more saturated than at sea-level (Doolette and Mitchell, 2001). As a result, when decompressing, such as during ascent from a dive, rapid ascent to altitude, or during extravehicular activity (EVA) in space, the tissues become supersaturated as the partial pressure of nitrogen exceeds the ambient partial pressure (Mitchell et al., 2022). When this happens bubbles can form as the gas expands, which can cause complications (Junes et al., 2022). Bubble formation leads to a range of symptoms including musculoskeletal pain, neurological impairment, and respiratory distress; collectively known as DCS (Mitchell et al., 2022), which poses a severe occupational risk to commercial and scientific divers (Dardeau et al., 2012), as well as aviators and astronauts (Conkin et al., 2017). Despite this, knowledge of how DCS manifests is largely unknown, and is a product of retrospective and empirical analysis conducted after individuals are diagnosed with DCS. Recently we have published new methods that track the movement of nitrogen in hyperbaric conditions; which could be vital to understanding how DCS forms, how it varies between individuals, and how it can be treated (Ashworth et al., 2024a; Ashworth et al., 2024b). These methods could be used to quantify the effectiveness of a prebreathe protocol prior to EVAs, and therefore can help optimize the effectiveness and efficiency of the prebreathe protocol. This would minimize the risk to astronauts and optimize time for mission objectives.

These methods have used the radionuclide nitrogen-13 (^13^N_2_). ^13^N_2_ is an isotope of nitrogen that emits beta-radiation with a half-life of 9.965 min. Beta-radiation can be tracked with positron-emission tomography (PET) to provide localized information as to where the radiation is coming from, enabling the tracking of nitrogen as ^13^N_2_ (Berger, 2003). Furthermore, the emitted positron rapidly annihilates with an electron, leading to the production of two gamma rays (Richardson, 1938). These gamma rays can be measured using a gamma counter which measure smaller quantities of radioactivity in smaller volumes (Wilde and Ottewell, 1980). Several studies have previously looked at ^13^N_2_ in humans, primarily injected as part of a saline solution to investigate lung function (Winkler et al., 2022; Vidal Melo et al., 2003). One study in humans breathing ^13^N_2_ gas in normobaric conditions with a gamma detector placed by the knee showed it was capable of being used to track nitrogen wash-in and wash-out (Weathersby et al., 1986), however it was not able to discern whether the reported nitrogen was in blood or tissue. Since this lone experiment ∼40 years ago, technical developments have produced new imaging modalities, such as PET scanning, which can provide spatial and temporal resolution of radioactive substances within the body thereby increasing the range of possibilities for tracking ^13^N_2_ movement through the body. Recently, we utilized these technological advancements to demonstrate firstly organ uptake of ^13^N_2_ in differing prebreathe conditions using gamma counting alone (Ashworth et al., 2024b). Secondly, we demonstrated the potential use of PET to provide spatial and temporal information on when ^13^N_2_ is *in vivo*, under hyperbaric conditions (Ashworth et al., 2024b). Both experiments showcase the potential for the use of ^13^N_2_ to research the mechanisms and potential treatments and prevention strategies relating to DCS. To further enable investigations on the effects of gas uptake and off-gassing we hypothesized that the method would work under normobaric conditions, and still detect nitrogen wash-in and wash-out (despite the much lower partial pressure of gas).

## 2 Methods

### 2.1 Use and care of animals

The animal used in this experiment was used in line with the UCSD standard of care, and under approval of the UCSD Institutional Animal Care and Use Committee (IACUC) protocol S19154. The animal was maintained under the surveillance of a veterinarian prior to being used in the study.

### 2.2 Nitrogen-13

The ^13^N_2_ was created off-site (PETNET, Siemens Medical Solutions, San Diego, CA) using a cyclotron in accordance with prior studies (Iwata et al., 1978). A liquid target containing aqueous NH_4_Cl solution (1.0 M, pH = 11) was irradiated with 15 MeV protons for 30 min ^13^N_2_ was extracted from the target by a helium sweep gas, which passed through a P_2_O_5_ absorber to purify the gas of NH_3_ and water vapor. The products were released into a vial, placed inside a lead ingot and casing, and delivered to the laboratory (transit time ∼10 min).

### 2.3 Analysis of ^13^N_2_

To confirm the presence of ^13^N_2_ several investigations were conducted on the produced gas containing ^13^N_2_. A syringe containing the gaseous ^13^N_2_ sample was placed in a dose calibrator (CRC-15W, Capintec, NJ, United States) with recordings were made every minute for 20 min. A separate sample of the gaseous ^13^N_2_ was placed in a gamma counter (2,480 Wizard^2^, Perkin Elmer, MA, United States) to obtain radioactive spectroscopy.

### 2.4 *In vivo* experiment

**FIGURE 1:**
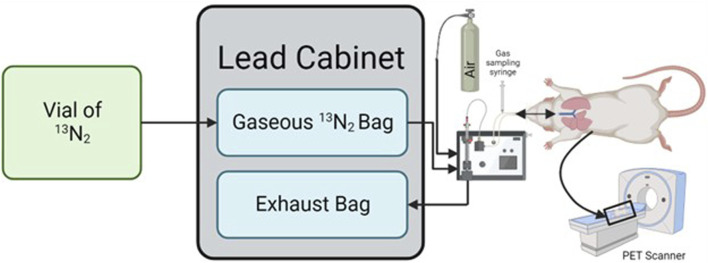
Schematic detailing how the process required to ventilate a rat with ^13^N_2_. The ^13^N_2_ arrives in liquid form and is bubbled into gaseous form which is collected in a non-diffusing bag housed within a lead cabinet. Gas is then drawn from this bag by a ventilator to a rat located inside a PET scanner, with the expired gas being collected in an exhaust bag within the lead cabinet. Created in BioRender.com.

Upon arrival at the laboratory the vial was placed into a dose calibrator and radioactivity recorded. The vial was then shaken for 10 s and bubbled (to release the ^13^N_2_ gas from solution) at 0.5 L min^-1^ directly from the isoflurane vaporizer (VS1482, Visual Sonics, Canada) into a non-diffusing bag (Figure 1 – Gaseous ^13^N_2_ Bag; 112,110, Hans Rudolph Inc., KS, United States) pre-filled with 7 L of oxygen-isoflurane mix (3% isoflurane). Bubbling continued for 1.5 min, causing flow of an additional 1.5 L of air into the bag, providing a total of ∼8.5 L which was estimated to be required for the 30 min scan time (30 min × 3.2 mL.breath^-1^ x 90 breath.min^−1^).

A Sprague-Dawley rat (6 months, 320 g) was anaesthetized using 5% isoflurane and placed on an intubation rack (Kent Scientific, CT, United States) in accordance with institutional review board approval (IACUC, UCSD, protocol S19154). With the airway exposed an intubation tube (16G Safelet Catheter, Exel, CA, United States) with an intubation safety wedge (Kent Scientific, CT, United States) was placed down the trachea. The intubation line was then connected to a mechanical ventilator (PhysioSuite, Kent Scientific, CT, United States) set to deliver air at a rate of 90 breaths per minute, at a tidal volume of 1% body mass (i.e., 300 g = 3 mL). Isoflurane was then reduced to 3% and the rodent was set on the PET scanner (eXplore VISTA DR, GE Healthcare, IL, United States) gantry, with the center of the PET located at the level of the lungs. The PET scanner recorded ^13^N_2_ using a dynamic emission scan for 30 min with energy windows of 250–750 keV. During the first 2 min of the PET scan the rat breathed oxygen, before being switched to ^13^N_2_ at 2 min. After 22 min of scanning had elapsed the inspiration line was switched back to the oxygen.

After 5 min, and thereafter at 10 min intervals, a gas sample was obtained from the inspiration line connected to the ventilator using a 10 mL gas syringe to determine the dose being delivered to the animal (Figure 1). The syringe was immediately placed in the dose calibrator, recorded, and then flushed into an extraction vent. Immediately prior to the gas sample collection a background radiation measurement was taken to be subtracted from the recorded values. These values were then corrected for the ^13^N_2_ half-life using Equation 1.

$$A_{0}=\frac{A\left(t\right)}{e^{-\lambda \cdot t}}$$ (1)

where $A_{0}$ is baseline counts per minute, $A\left(t\right)$ is counts per minute at the time point $\left(t\right)$ , and λ is equal to 0.693/9.965 min, where 0.693 is equal to the natural logarithm of 2, and 9.965 min is the half-life of ^13^N_2_.

Upon cessation of the PET scan the rodent was surgically opened and euthanized by a mixed blood draw from the heart. Immediately thereafter, the liver, brain, femur and quadriceps muscles were surgically removed and, alongside the remaining rat, were placed in the dose calibrator to assess whole-rat radioactivity. The organs were then placed into a gamma counter (Gamma 8,000, Beckman, IN, United States) to obtain organ-specific counts. All counts were obtained within a window of 400–600 keV. Counting continued until the certainty reached 95%, or 10 min had elapsed. Organ counts were corrected firstly by subtracting background radiation, and then using Equation 1 to account for the difference in time between each sample, effectively reporting the counts when the counting process began. All organs were then weighed (CP64, Sartorius, Germany) to enable calculation of counts relative to mass.

The PET image was analyzed in Fiji image analysis software (Schindelin et al., 2012) in composite images of 2 min each. Images were converted into 3D stacks, and the entire image in each view had signal intensity recorded. Mean and standard deviation (SD) were calculated for each 2 min block. Additionally, the PET images for minutes 2-22 were amalgamated into one image to visualize the ^13^N_2_ activity in the lung.

## 3 Results

**FIGURE 2:**
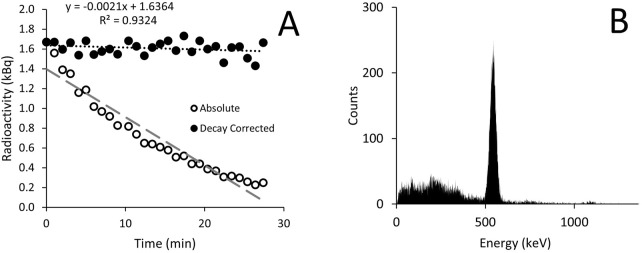
Radioactivity in the gaseous ^13^N_2_ recorded every minute using a dose calibrator **(A)** and radioactive spectroscopy measured using a gamma counter **(B)**. Results are displayed as both absolute values (open circles) and values adjusted for ^13^N_2_ radioactive decay (closed circles). The spectroscopy shows a peak just above 500 keV.

The vial produced contained 1,158.1 MBq, which had reduced to 577.2 MBq by the time it was measured in the laboratory. Analysis of the gaseous ^13^N_2_ gas showed decay as expected (Figure 2A) with good reliability. The spectroscopy revealed a peak just over 500 keV, close to the expected peak for positron emission of 511 keV (Figure 2B).

The bubbling of the vial contents left 418.1 MBq in the vial, indicating that ∼75.1 MBq of the original dose (when corrected) had been removed from the vial as ^13^N_2_ and entered the gaseous ^13^N_2_ bag. The in-line gas concentration was consistent between the two measurement periods, and was reduced when the ^13^N_2_ flow stopped (Table 1).

**TABLE 1:** Radioactivity of inspiration line gas samples while breathing^13^N_2_ gas. Samples are corrected for radioactive decay.^13^N_2_ gas was turned off after 22 min.

Time (min)	Gas radioactivity (kBq.ml^-1^)
5	5.735
15	5.254
25	0.148

**FIGURE 3:**
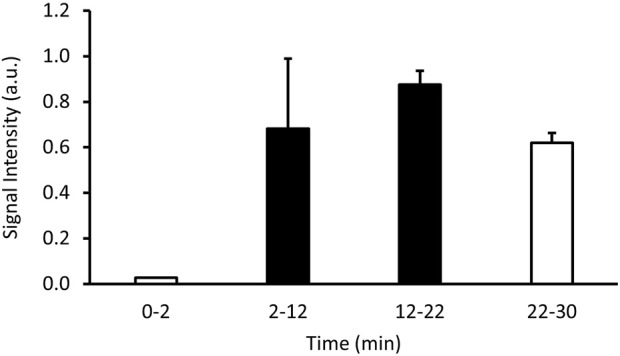
Signal intensity in arbitrary units (a.u.) over time showing the changes in radioactive positron emission from a sedated rodent breathing ^13^N_2_ gas. Values shown are an average signal intensity of the specified interval. An oxygen-isoflurane mix was breathed from 0 to 2 and 22–30 min (white bars), while the ^13^N_2_ gas was turned on from 2 to 22 min (black bars).

**FIGURE 4:**
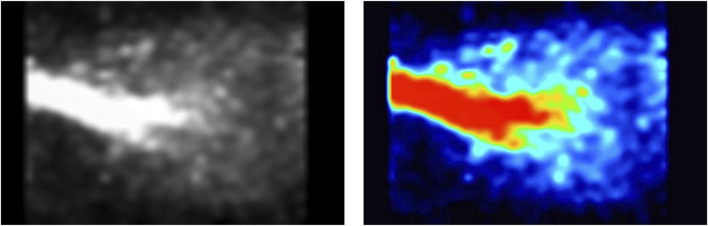
PET image of rat lung while breathing ^13^N_2_ gas. Recording window was set to 250–750 keV. The trachea conducts all of the radioactive gas, and therefore has the strongest signal (red). The radioactivity then spreads throughout the lungs, being more condensed within the middle of the lungs as the lungs are continuously moving due to respiration.

The signal intensity recorded by the PET increased once the ^13^N_2_ was connected and approached a plateau before reducing in the final minutes of the experiment (Figure 3). Visual analysis of the PET image reconstruction clearly shows the shape of the lungs being filled with ^13^N_2_, seen alongside the intubation tube and trachea which show the highest concentrations of positron emission (Figure 4).

Upon conclusion of the PET scan the whole rat had 58.1 kBq of radioactivity. The liver, brain and bone had higher relative counts per minute than the blood, whereas the muscle had lower counts (Table 2).

**TABLE 2:** Organ parameters following breathing^13^N_2_ for 20 min followed by 10 min of breathing oxygen. All counts were corrected for radioactive decay (Equation 1).

Sample	Mass (g)	Error (%)	Counts.min^−1^.g^−1^
Blood	3.722	1.99	3,390.28
Liver	8.751	1.91	12,593.29
Brain	1.811	1.99	5,382.77
Muscle	1.364	1.99	2,686.57
Bone	0.180	4.53	3,882.01

## 4 Discussion

This study successfully demonstrated a method of tracking nitrogen gas through the body that could be imaged both *in vivo* using PET, where ^13^N_2_ was tracked going in and out of the lung, and *ex vivo* where a gamma counter assessed individual organs.

The total radioactive volume received was only 577.2 MBq, a safe experimental quantity, that comfortably lasted the duration of the experiment (∼1 h). Future experiments could likely be of longer duration or increase the starting dose. The radioactive gas had both the decay constant, and decay spectroscopy of ^13^N_2_ (Figure 2), thereby confirming the radioactive substance breathed was ^13^N_2_. The supply of ^13^N_2_ to the rat measured in the inspiratory line was reasonably consistent between the two measurements taken during ^13^N_2_ delivery (Table 1) suggesting good mixing of gas within the gaseous ^13^N_2_ bag. The constant supply of ^13^N_2_ increases the potential for uptake over time as only a small volume of ^13^N_2_ will be taken up with each breath, whereas if a bolus of ^13^N_2_ was delivered a large proportion could be expired.

The PET image shows ^13^N_2_ in the main areas we would expect to see it–the trachea and the lungs, with minor signal coming from the surrounding areas (Figure 4). During the first 2 min of the experiment while oxygen-isoflurane was breathed PET signal resembled background levels (Figure 3). Upon switching to ^13^N_2_ the PET signal increased steadily from 2 to 12 min, reaching a plateau from 12 to 22 min, suggesting equilibrium of pulmonary ^13^N_2_ was reached rapidly (Figure 3). Then when ^13^N_2_ delivery ceased we saw a drop in PET signal (Figure 3), showing the washout of ^13^N_2_ from the lung and surrounding tissue. Due to the natural movement of the lungs during image acquisition it is hard to quantify the amount of ^13^N_2_ taken up in pulmonary tissue or adjacent tissue, but the subsequent organ counts showed that ^13^N_2_ uptake was widespread.

The organ counts showed higher relative ^13^N_2_ content in the liver, brain and bone than the blood (Table 2), which despite being only for a single rat, is in line with current physiological understanding. Although these results do differ from those observed in our prior study (Ashworth et al., 2024b), this can be attributed to differences in the prebreathe procedure and post-breathing oxygen in the present study enabling off-gassing. The brain has the highest proportion of fat of any organ and is highly perfused, so might be expected to take up ^13^N_2_ more than the other organs (O'Brien and Sampson, 1965). However, these factors also mean that it will off-gas faster during the oxygen breathing period of the experiment. Indeed, neurological decompression sickness is largely reported during short dives (Schipke and Tetzlaff, 2016; Lang et al., 2013), suggestive that brain is a tissue with fast nitrogen kinetics. A review of solubility coefficients by Weathersby and Homer (Weathersby and Homer, 1980) showed brain and blood to have similar levels of nitrogen, with a range of approximately 50%–150%. In the current study the brain has 159% more ^13^N_2_ than the blood, falling just outside of this range, which may be due to the breathing gas being oxygen which would increase the potential for nitrogen uptake. Only one study used the same method to measure brain and blood nitrogen content, finding brain tissue to be 13% more soluble than blood in rabbits (Ohta et al., 1979). It is unclear whether using a perfused brain, as in the current study, would lead to similar results, and to what degree organ perfusion can be quantified. The liver is also highly perfused, and is the fattiest abdominal organ, which likely accounts for the large values seen here (Sijens et al., 2010). Bone ^13^N_2_ content was also elevated compared to blood, although only slightly. This could be due to bone marrow making up 10% of adult human total fat content which may help with nitrogen uptake. Conversely, muscle has less fat, with human intramuscular thigh fat as low as 8% of the tissue, and therefore would be expected to store less ^13^N_2_ (Goodpaster et al., 2000). While the present study looked at a single rodent, the muscle was observed to store less ^13^N_2_ than blood (Table 2).

### 4.1 Methodological limitations

The use of a PET scanner alone makes tracking the exact organs taking up ^13^N_2_ difficult as organ positions vary between individuals, the PET provides no anatomical landmarks, and the spatial resolution of PET is low (Vaquero and Kinahan, 2015). For the lungs which receive a large dose, their outline is easily observed due to the contrast with adjacent tissue (Figure 4), but this is not the case for other tissues. It is possible that future studies could look to use PET and computed tomography in concert to guide locating where ^13^N_2_ is, particularly in studies where survival is prioritized (Vaquero and Kinahan, 2015). In non-survival experiments, the use of the gamma counter provides a unique opportunity to gather rich data as this is sensitive to much lower radiation doses.

However, the 10 min half-life of ^13^N_2_ still limits experiments to be short (∼100 min) as otherwise the radioactive signal dissipates to that of background radiation. This limits the scope of experiments that can be done with this method while keeping radiation within safe limits. However, continuous generation and supply of ^13^N_2_ could provide a mechanism by which experimental times could be extended. Alternatively, ^13^N_2_ could be supplied at any point within the experimental procedure, thereby evaluating different stages during the nitrogen wash-in/wash-out process.

Usually a standardized uptake value is used to quantify PET signal (Kinahan and Fletcher, 2010), which could be achieved in the future using biopsies or a blood draw to calibrate the signal.

### 4.2 Future applications

The current experiment was performed at normobaric pressure, with enough signal to noise without the compression and subsequent decompression that occurs with diving. This shows that the method could be used to optimize oxygen prebreathing protocols before EVAs in spaceflight. Using larger animals and combining the PET with a CT scan in the future would enable full body scans and give strong spatial resolution to determine exactly which organs are most at risk following a prebreathe procedure. Indeed, while this experimental paradigm has been devised for rodents it can be extended to pigs and possibly humans in the future. Application of this technique to humans would also require careful management of the radiation dose. The dose would need to be small enough to cause minimal harm to the participant, whilst remaining large enough to provide the spatial resolution needed for robust scientific data.

## Data Availability

The raw data supporting the conclusions of this article will be made available by the authors, without undue reservation.
